# Fire needling therapy for neurodermatitis: a systematic review and meta-analysis of randomized controlled trials

**DOI:** 10.3389/fmed.2025.1639713

**Published:** 2025-11-20

**Authors:** Xiao Qiu, Xiangyu Hu, Ruiling Jia, Yuting Ding, Yuyi Wang

**Affiliations:** 1 Department of Dermatology, The First Affiliated Hospital of Chongqing College of Traditional Chinese Medicine, Chongqing, China; 2 Department of Dermatology, Chongqing Hospital of Traditional Chinese Medicine, Chongqing, China; 3 Chongqing Key Laboratory of Integrative Dermatology Research, Chongqing, China; 4 Chongqing Clinical Research Center for Dermatology, Chongqing, China; 5 Chongqing Yubei District Hospital of Traditional Chinese Medicine, Chongqing, China

**Keywords:** fire needling therapy, neurodermatitis, lichen simplex chronicus, meta-analysis, traditional Chinese medicine

## Abstract

**Objective:**

This meta-analysis aimed to evaluate the clinical effectiveness and safety of fire needling therapy for neurodermatitis, either as monotherapy or in combination with conventional treatments.

**Methods:**

Following PRISMA 2020 guidelines, randomized controlled trials were systematically searched across Chinese and English databases up to October 2024. Ten RCTs involving 868 patients were included. Outcomes assessed included effectiveness rate, recurrence rate, pruritus scores, inflammatory cytokine levels, Dermatology Life Quality Index (DLQI), and adverse events. Data were analyzed using RevMan 5.4.1, with effect sizes expressed as risk ratios or mean differences.

**Results:**

Fire needling monotherapy showed no significant superiority over conventional treatment in effectiveness rates (*p* > 0.05). However, combined therapy demonstrated significantly higher effectiveness at the >90% threshold after 4 weeks (RR: 2.04, 95% CI: 1.61–2.59) and reduced recurrence rates within 6 months after 2-week treatment (RR: 0.36, 95% CI: 0.14–0.93). Combined therapy also improved DLQI scores (MD: −3.91, 95% CI: −6.15 to −1.67) and reduced pruritus (MD: −0.25, 95% CI: −0.43 to −0.07) and inflammatory markers (TNF-α, IL-4, IL-6, IL-8, IgE; *p*  0.05). Adverse events were mild.

**Conclusion:**

Fire needling therapy combined with conventional treatment may enhance therapeutic outcomes for neurodermatitis, particularly in reducing pruritus, improving quality of life, and modulating inflammation. However, limitations include methodological flaws in included studies and regional publication bias. Higher-quality RCTs are needed to validate these findings and clarify long-term effectiveness.

**Systematic review registration:**

https://www.crd.york.ac.uk/PROSPERO/view/CRD42024617150, identifier CRD42024617150.

## 1 Introduction

Neurodermatitis, also known as lichen simplex chronicus, is a common chronic skin disorder characterized by paroxysmal intense itching and lichenified skin lesions (1, 2). The condition predominantly occurs in areas prone to friction, such as the neck, elbows, and lumbosacral region, and can significantly impair patients’ quality of life. According to epidemiological data, the estimated global incidence of neurodermatitis is approximately 1%–2%, with some regional studies indicating an upward trend (3). The disease is more prevalent among young and middle-aged adults, and certain studies suggest a slightly higher incidence in women than in men (4). The etiology of neurodermatitis is complex and involves multiple factors. Firstly, neuropsychiatric factors play a critical role in its pathogenesis (5). Research indicates that prolonged psychological stress, anxiety, and depression can disrupt the neuroendocrine system, thereby triggering or exacerbating neurodermatitis (6, 7). Secondly, immune dysregulation is another key mechanism underlying the disease (8). Studies have found that patients with chronic inflammatory skin conditions often exhibit overactivation of Th2-type immune responses in the skin (9), leading to abnormal expression of inflammatory cytokines such as IL-4, IL-6, and IL-13, which in turn provoke skin inflammation and itching (10). Additionally, impaired skin barrier function is a hallmark of chronic inflammatory skin disorders (11). The compromised skin barrier allows external irritants to penetrate more easily, further aggravating inflammation and itching (12). Currently, the treatment of neurodermatitis faces numerous challenges. Conventional therapies primarily include topical corticosteroids, calcineurin inhibitors, and antihistamines (13). In clinical practice and prior research, these methods are often used to address the disease’s multi-faceted symptoms: for example, topical medium-potency corticosteroids serve as first-line options for controlling local inflammation and lichenified lesions, while oral antihistamines are commonly adjunctive to relieve pruritus—yet even this combined regimen fails to achieve sustained efficacy, with over half of patients experiencing symptom recurrence within 6 months (14). Moreover, long-term use of corticosteroids and calcineurin inhibitorsmay also cause side effects such as skin atrophy and telangiectasia (15). Consequently, identifying a safe, effective, and minimally adverse treatment approach has become a focal point of current research (16). In recent years, fire needling therapy, a traditional Chinese medical treatment, has demonstrated unique advantages in managing eurodermatitis (17). Combining the effects of acupuncture and heat moxibustion, fire needling therapy applies thermal stimulation to local lesions to enhance the body’s energy for disease treatment (18). Modern research suggests that fire needling therapy may exert therapeutic effects through multiple pathways. Firstly, it can inhibit neurogenic inflammation, reducing the release of inflammatory mediators and thereby alleviating itching and inflammatory responses (19). Secondly, it modulates immune responses by suppressing the overactivation of Th2-type immunity and lowering levels of inflammatory cytokines such as IL-6 and TNF-α (20). Therefore, this study aims to systematically evaluate the clinical effectiveness of fire needling therapy for neurodermatitis through a meta-analysis, with the goal of providing more evidence-based medical support for its treatment and offering references for the clinical application of fire needling therapy.

## 2 Materials and methods

This meta-analysis was conducted in accordance with the Preferred Reporting Items for Systematic Reviews and Meta-Analyses (PRISMA) 2020 guidelines (21) and followed the methodological standards outlined in the Cochrane Handbook for Systematic Reviews of Interventions (22). The study protocol was prospectively registered with PROSPERO (registration number: CRD42024617150).

### 2.1 Search strategy

The search was conducted across multiple databases, including China National Knowledge Infrastructure (CNKI), VIP Chinese Science and Technology Journal Database, Wanfang Database, China Biology Medicine Database (Sinomed), PubMed, and Cochrane Library, with the search period extending up to October 2024. The Chinese search terms were “火针,““火疗,” “烧针,” “神经性皮炎,” “慢性单纯性苔藓,” “临床,” and “随机”; English search terms were “fire needling,” “neurodermatitis,” “lichen simplex chronicus,” “clinical,” “random.” These search terms were used as keywords for a logical combination of searches in the subject, title, abstract, and full-text fields. The specific search strategy for each database can be found in Supplementary Table 1.

### 2.2 Inclusion and exclusion criteria

#### 2.2.1 Inclusion criteria

(1) Study type: Randomized controlled trials (RCTs);
(2) Participants: Patients with a confirmed diagnosis of neurodermatitis, regardless of age or disease stage;
(3) Interventions: The treatment group received fire needling therapy alone or combined with conventional treatment (e.g., topical corticosteroids or oral antihistamines), while the control group received conventional treatment alone, with consistent treatment duration between groups;
(4) Outcome measures: Primary outcomes included effectiveness rate [treatment response thresholds were predefined by the relative reduction in Pruritus Visual Analogue Scale (VAS) scores and lesion severity scores from baseline, with specific definitions: >30% improvement (30%–69% reduction vs. baseline), >70% improvement (70%–89% reduction vs. baseline), and >90% improvement (≥90% reduction vs. baseline)] and recurrence rate; secondary outcomes comprised pruritus scores, inflammatory cytokine levels, Dermatology Life Quality Index (DLQI), and adverse events.

#### 2.2.2 Exclusion criteria

(1) Duplicate publications;
(2) Studies with only abstracts available but no full text;
(3) Studies with incomplete data.

### 2.3 Literature screening

(1) Initial screening: Bibliographic records were imported into Noteexpress 4.1, duplicate records between databases were eliminated, then abstracts were read and preliminary screening was conducted based on inclusion and exclusion criteria;
(2) For records where inclusion could not be determined from the abstract and further evaluation was needed, the full text was downloaded;
(3) The full text was read and, according to the study’s inclusion criteria, the final determination of which studies could be included was made.

### 2.4 Data extraction

A standardized extraction form was created using Excel 2013 to collect:

(1) Basic study characteristics.
(2) Demographic features of participants.
(3) Risk of bias items for RCTs.
(4) Intervention and control protocols.
(5) Outcome measures.

### 2.5 Methodological quality assessment

The Cochrane Risk of Bias Tool was used to evaluate methodological quality across seven domains: random sequence generation, allocation concealment, blinding of participants and personnel, blinding of outcome assessment, incomplete outcome data, selective reporting, other biases. Each domain was rated as “low risk,” “unclear risk,” or “high risk” according to Cochrane standards (22). All data extraction and methodological assessments were first performed by one researcher and then cross-checked by another, with any discrepancies resolved through consensus discussion.

### 2.6 Statistical processing

Meta-analysis was performed using Revman5.4.1 software provided by the Cochrane Collaboration for trials with identical or similar experimental and control interventions. For measurement data, mean difference (MD) and 95% confidence interval (95% CI) were used to evaluate the effect; for count data, relative risk (RR) and its 95% CI were used for effect evaluation. The *I*^2^ statistic was used to assess the magnitude of statistical heterogeneity among included studies. When there was no heterogeneity or minor heterogeneity among studies (*I*^2^ ≤ 25%), a fixed-effects model was used; when there was greater heterogeneity (25% < *I*^2^ ≤ 75%) but no obvious clinical heterogeneity, a random-effects model was adopted; if heterogeneity was particularly high (*I*^2^ > 75%), quantitative data merging was not performed, and only individual study results were described. When obvious clinical heterogeneity existed, the source of heterogeneity was analyzed and subgroup analysis was conducted.

## 3 Results

### 3.1 Literature search and screening results

**Figure 1:**
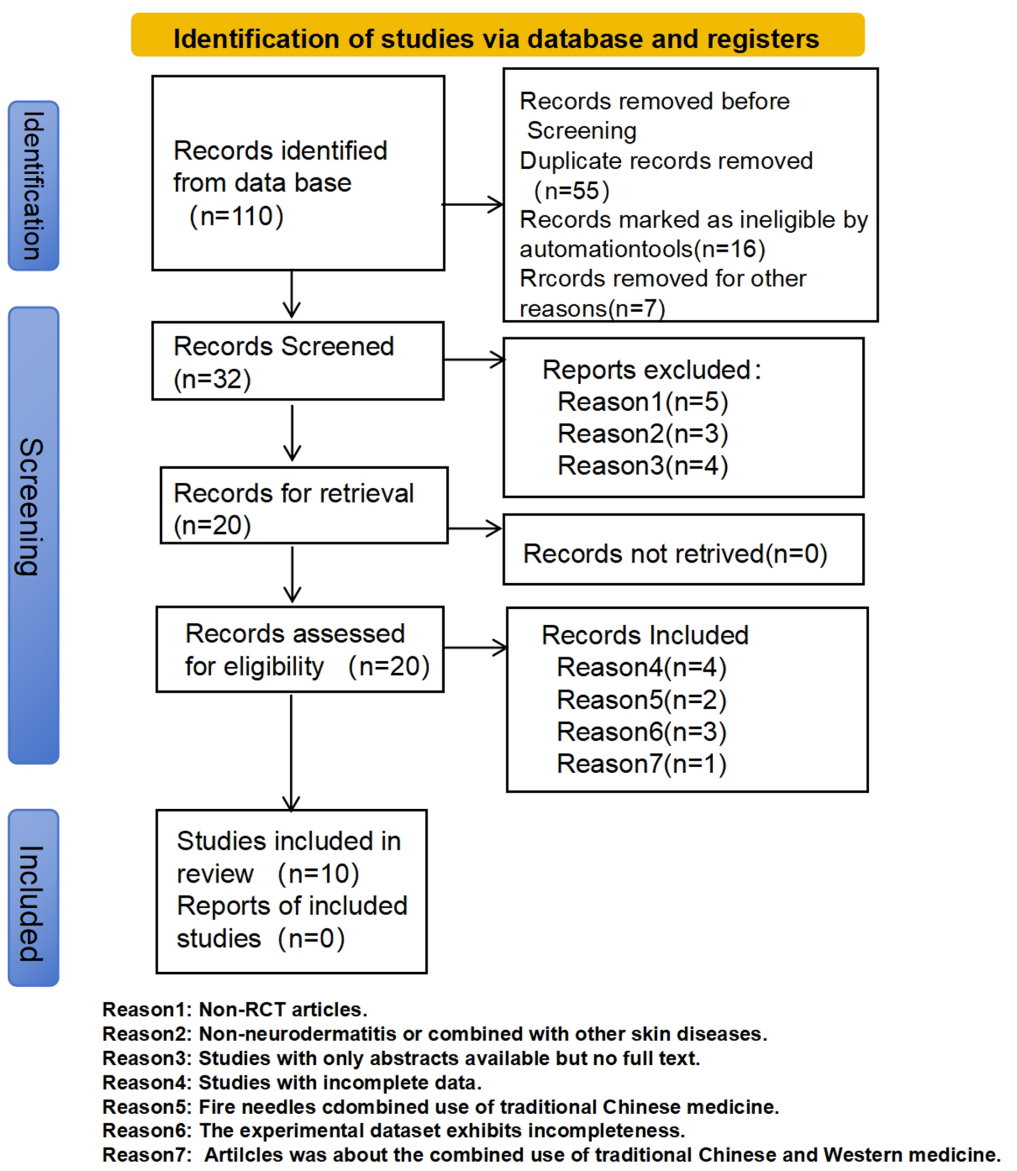
PRISMA flow diagram for literature selection. Reason 1: Non-RCT articles. Reason 2: Non-neurodermatitis or combined with other skin diseases. Reason 3: Studies with only abstracts available but no full text. Reason 4: Studies with incomplete data. Reason 5: Fire needles combined use of traditional Chinese medicine. Reason 6: The experimental dataset exhibits incompleteness. Reason 7: Articles was about the combined use of traditional Chinese and Western medicine.

Six major Chinese and English databases were searched, yielding 110 records in total. After deduplication, initial screening, full-text reading, and other procedures, 10 RCTs (23–32) meeting the inclusion and exclusion criteria were finally included. The specific process is shown in Figure 1.

### 3.2 Characteristics of included studies

A total of 10 studies were included, involving 10 RCTs (23–32) and 868 patients with neurodermatitis. All studies were conducted in China and published in Chinese journals. Among them, seven studies (24–30) compared fire needling therapy combined with conventional treatment to conventional treatment alone, while three studies (23–25) compared fire needling therapy alone to conventional treatment alone. The treatment duration ranged from 2 to 4 weeks, with detailed information provided in Table 1.

**Table 1:** Characteristics of included studies.

Study ID	No. (M/F)		Mean age (y)		Disease (y)	course	Intervention	Control	Treatment course	Outcomes
	T	C	T	C	T	C				
Wang (26)	33/27	35/25	46.8	45.4	6.5	6.7	Fire needing therapy combined with oral loratadine. Fire needling therapy: The tip of a filiform needle is heated until red-hot and then quickly inserted into the lesion. For mild lesions, the needle is inserted to the base of the skin. For thicker lesions or those with severe itching, the insertion depth is adjusted according to the degree of thickness, administered twice a week. Oral loratadine: 5 mg/day.	Oral loratadine: 5 mg/day.	4 weeks	Effectiveness rate, inflammatory factors (IL-4, IgE), histamine changes, DLQI
Zhang et al. (30)	22/28	24/26	55	57	1	0.83	Fire needling therapy combined with topical halometasone Cream. Fire needling therapy: A filiform needle is heated until red-hot and then inserted into the lesion to a depth of 1–2 mm, reaching the base of the lesion, before being swiftly withdrawn. The insertion depth is adjusted based on lesion thickness. The puncture spacing is 2–3 mm. Administered once weekly. Topical halometasone cream: applied twice daily.	Halometason e cream for topical use, twice daily.	4 weeks	Effectiveness rate, pruritus score
Ming and Xie (32)	24/36		39.4		7.3	7.3	Fire needling therapy combined with oral loratadine treatment. The fire needles were inserted to a depth of 0.2–0.5 cm, spaced 0.3–0.4 cm apart, administered twice weekly. Oral loratadine tablets (10 mg) were given once daily.	Oral loratadine: 10 mg/day.	2 weeks	Effectiveness rate, recurrence rate
Yu and Chai (28)	25/ 15	27/ 13	30.6	30.8	2.3	2.4	Fire needle therapy combined with topical halometasone cream application. The fire needle treatment was performed twice weekly, and halometasone cream was applied once daily.	Halometason e cream for topical use, once daily.	4 weeks	Effectiveness rate
Zhao et al. (29)	24/29	32/21	37.01	38.13	2.49	2.53	Fire needling therapy combined with halometasone ointment for treatment. The fire needles were vertically inserted from the edge of the lesion toward the center, with a depth of 0.2–0.5 cm and an interval of 0.2–0.5 cm between adjacent needles. For thicker lesions, the depth could be increased and the needle spacing reduced. The treatment was performed twice weekly. Halometasone ointment was applied topically once daily.	Halometason e cream for topical use, once daily.	4 weeks	Effectiveness rate, inflammatory factors (IL-6, IL-4), DLQI, adverse events, recurrence rate
Wang et al. (31)	34/32	35/31	35	36	12.56	12.34	Fire needling therapy combined with oral loratadine for treatment. The fire needles were inserted vertically and rapidly into the affected skin to a depth of 1–2 mm with an approximate spacing of 1 cm. The needling was performed progressively from the edge of the lesion toward the center, with slightly denser punctures in areas of thickened skin. Treatment was administered once every 5 days. Oral loratadine tablets (10mg) were given once daily.	Oral loratadine: 10 mg/day.	4 weeks	Effectiveness rate, DLQI, inflammatory factors (TNF-α, IL-6, IL-8), pruritus score, histamine changes
You et al. (27)	35/45	33/37	35.40	35.51	1.1	1.1	Fire needling therapy combined with topical compound clobetasol propionate ointment for treatment. The fire needles were inserted vertically into the affected skin to a depth of approximately 1–2 mm, with an inter-needle spacing of about 0.5 cm. The treatment was administered twice weekly. Compound clobetasol propionate ointment was applied topically twice daily.	Compound clobetasol propionate ointment was applied topically twice daily.	4 weeks	Effectiveness rate, adverse events
Zhu (23)	18/ 17	16/ 19	39.98	39.56	2.8	2.86	Fire needle therapy was applied to the lesional area with vertical punctures at a depth of approximately 1-2 mm and a frequency of 0.5 punctures per second. The needles were spaced 0.5-1 cm apart, progressing gradually from the lesion periphery toward the center, with slightly denser punctures in areas of significant	Halometason e cream for topical use, twice daily.	4 weeks	Effectiveness rate, pruritus score, adverse events, recurrence rate
Li et al. (25)	17/13	20/10	36.98	38.66	3.2	2.9	The fire needles were applied progressively from the periphery towards the center of the lesion, with slightly denser punctures in areas of significant thickening. Treatment was administered twice weekly.	Triamcinolone acetonide and urea ointment for topical application, twice daily.	4 weeks	Effectiveness rate
Tian (24)	32/38		Unclear	Unclear	Unclear	Unclear	Fire needling therapy was performed with rapid punctures on the lesional area at approximately 2 mm depth and 5–10 mm spacing, progressing from the periphery toward the center of the lesion.	Triamcinolon e acetonide ointment for topical use, twice daily.	4 weeks	Effectiveness rate

DLQI, Dermatology Life Quality Index; IL-4, Interleukin-4; IL-6, Interleukin-6; IL-8, Interleukin-8; IGE, Immunoglobulin; T, treatment group; C, control group.

### 3.3 Methodological quality of included studies

**Figure 2:**
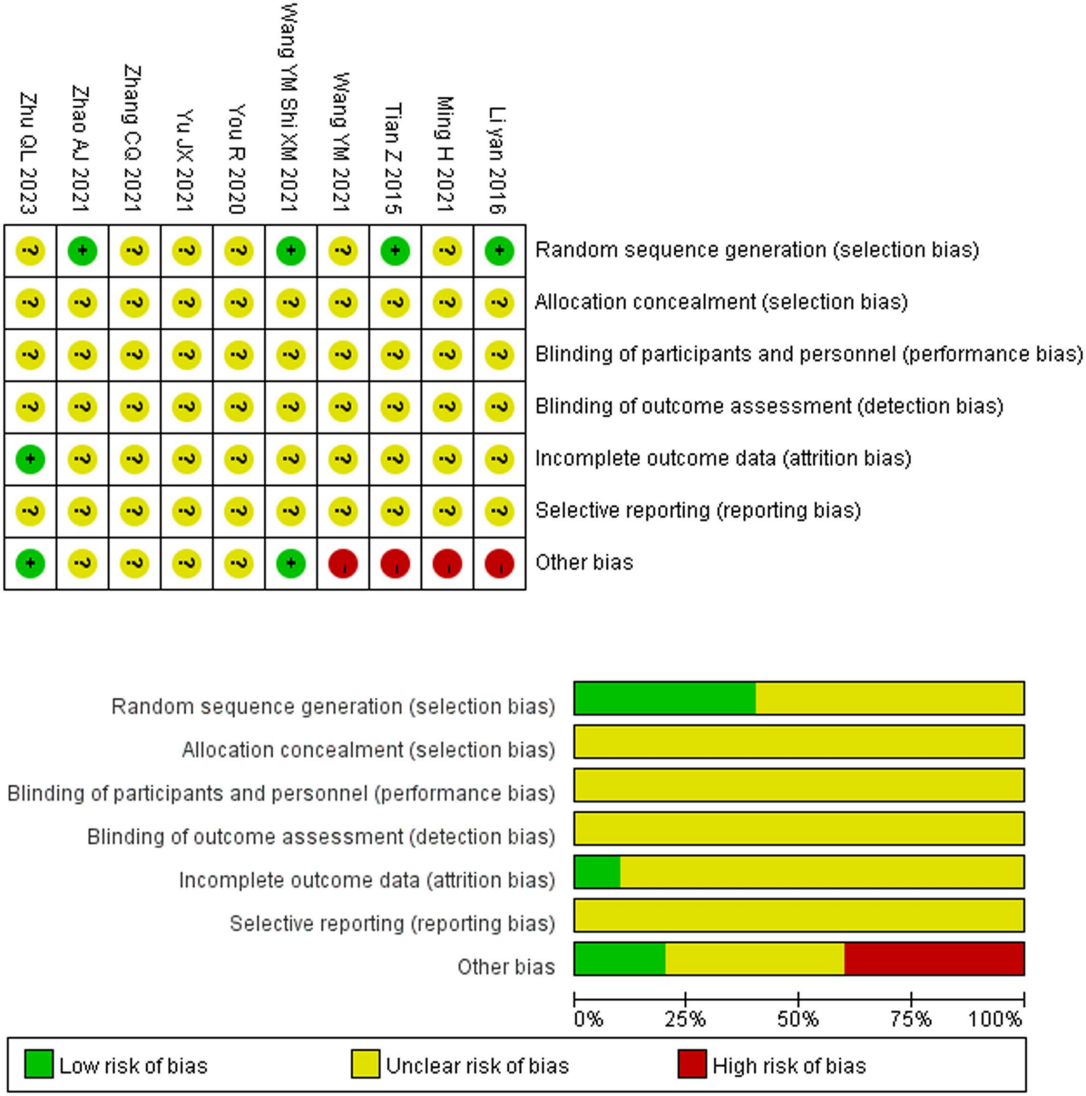
Methodological quality graph of included studies.

Among the 10 included RCTs (23–32), only four studies (24, 25, 29, 31) reported using methods such as random number tables for group allocation, while the remaining six studies (23, 26–28, 30, 32) did not describe the method of generating random sequences. None of the studies mentioned whether allocation concealment was applied. Since fire needling therapy and conventional treatment cannot be placebo-controlled, blinding of researchers and participants was not implemented in any of the studies, and no study reported whether outcome assessors were blinded. Only one study (24) documented patient dropouts. None of the studies had registered their research protocols, making it impossible to assess the risk of selective outcome reporting. Additionally, most studies exhibited other biases, such as the lack of sample size estimation. The methodological quality assessment of the included studies is presented in Figure 2.

### 3.4 Effectiveness outcomes

Table 2 showed the detailed effect estimates of fire needling therapy for neurodermatitis. Table 3 showed the detailed GRADE (Grading of Recommendations assessment) of fire needling therapy for neurodermatitis.

**Table 2:** Meta-analysis of the RCTs on fire needling therapy for neurodermatitis.

Comparisons	Outcomes	Number of studies	Number of participates	Included studies
Fire needling therapy vs. conventional treatment				
Effectiveness (>30%,4w)	RR:1.04 [0.90, 1.20]	3	200	(23–25)
Effectiveness (>70%,4w)	RR:1.07 [0.84, 1.37]	3	200	(23–25)
Effectiveness (>90%,4w)	RR:1.30 [0.66, 2.56]	3	200	(30–32)
Fire needling therapy combined with conventional treatment versus conventional treatment alone				
Effectiveness (>30%,2w)	RR:0.97 [0.86, 1.08]	1	60	(32)
Effectiveness (>70%,2w)	RR:1.00 [0.74, 1.36]	1	60	(32)
Effectiveness (>30%,4w)	RR:1.00 [0.74, 1.36]	5	608	(26, 27) (29–31)
Effectiveness (>70%,4w)	RR:1.42 [1.24, 1.63]	4	476	(26, 27) (30, 31)
Effectiveness (>90%,4w)	RR:1.89 [1.51, 2.37]	6	698	(26–31)
Recurrence rate (2w)	RR:0 0.36 [0.14, 0.93]	1	106	(32)
Recurrence rate (4w)	RR:0 0.33 [0 0.11, 1.03]	2	120	(23, 29)
DLQI (4w) MD:-3 0.91[−6 0.15, −1.67]		3	358	(26, 29, 31)
Histamine (4w) MD:-5.76[−14.94, 3.42]		2	126	(26, 31)
TNF-α (4w) MD:-5.66 [−8.81, −2.52]		2	179	(26, 31)
IGE (4w) MD:-1.23[−1.51,-0.95]		1	120	(26)
IL-4 (4w) MD:-1.41 [−2.02, −0.79]		2	226	(26, 29)
IL-6 (4w) MD:-9.34[−12.02,-6.77]		3	358	(26, 29, 31)
IL-8 (4w) MD:-6.10[−8.44,-3.76]		1	132	(31)
Itching score (4w) MD:-0 0.55 [1 0.02, −0.09]		3	276	(29, 30)

DLQI, Dermatology Life Quality Index; IL-4, Interleukin-4; IL-6, Interleukin-6; IL-8, Interleukin-8; IGE, Immunoglobulin E; MD, mean difference; RR, risk ratio.

**Table 3:** GRADE evidence summary table.

Outcomes	No. of patients		Effect		Certainty of the evidence (GRADE)
	Fire needling	Conventional treatment	Relative (95% CI)	Absolute (95% CI)	
Fire needling therapy vs. conventional treatment-effectiveness (>30%,4w)	91/100 (91.0%)	86/100 (86.0%)	RR 1.04 (0.90 to 1.20)	34 more per 1,000 (from 86 fewer to 172 more)	⨁⨁◯◯Low^a^^,^^b^^,^^c^^,^^e^
Fire needling therapy vs. conventional treatment-effectiveness (>70%,4w)	69/100 (69.0%)	63/100 (63.0%)	RR 1.07 (0.84 to 1.37)	44 more per 1,000 (from 101 fewer to 233 more)	⨁⨁◯◯ Low^a^^,^^b^^,^^c^^,^^e^
Fire needling therapy vs. conventional treatment-effectiveness (>90%,4w)	859/1225 (70.1%)	669/1205 (55.5%)	RR 1.22 (1.10 to 1.35)	122 more per 1,000 (from 56 more to 194 more)	⨁⨁◯◯ Low^a^^,^^b^^,^^c^^,^^d^^,^^e^
Fire needling therapy combined with conventional treatment versus conventional treatment -effectiveness (>30%,2w)	28/30 (93.3%)	29/30 (96.7%)	RR 0.97 (0.86 to 1.08)	29 fewer per 1,000 (from 135 fewer to 77 more)	⨁⨁◯◯ Low^a^^,^^c^^,^^e^^,^^f^
Fire needling therapy combined with conventional treatment versus conventional treatment-effectiveness (>70%,2w)	22/30 (73.3%)	22/30 (73.3%)	RR 1.00 (0.74 to 1.36)	0 fewer per 1,000 (from 191 fewer to 264 more)	⨁⨁◯◯ Low^a^^,^^c^^,^^e^^,^^f^
Fire needling therapy combined with conventional treatment versus conventional treatment-effectiveness (>90%,2w)	12/30 (40.0%)	16/30 (53.3%)	RR 0.75 (0.43 to 1.30)	133 fewer per 1,000 (from 304 fewer to 160 more)	⨁⨁◯◯ Low^a^^,^^c^^,^^e^^,^^f^
Fire needling therapy combined with conventional treatment versus conventional treatment-effectiveness (>30%,4w)	298/309 (96.4%)	248/299 (82.9%)	RR 1.16 (1.10 to 1.22)	133 more per 1,000 (from 83 more to 182 more)	⨁⨁◯◯ Low^a^^,^^b^^,^^c^^,^^e^
Fire needling therapy combined with conventional treatment versus conventional treatment-effectiveness (>70%,4w)	184/243 (75.7%)	124/233 (53.2%)	RR 1.42 (1.24 to 1.63)	224 more per 1,000 (from 128 more to 335 more)	⨁⨁◯◯ Low^a^^,^^b^^,^^c^^,^^e^
Fire needling therapy combined with conventional treatment versus conventional treatment-effectiveness (>90%,4w)	138/283 (48.8%)	68/283 (24.0%)	RR 2.04 (1.61 to 2.59)	250 more per 1,000 (from 147 more to 382 more)	⨁⨁◯◯ Low^a^^,^^b^^,^^e^
Recurrence rate (2w)	2/53 (3.8%)	8/53 (15.1%)	RR 0.36 (0.14 to 0.92)	97 fewer per 1,000 (from 130 fewer to 12 fewer)	⨁⨁◯◯ Low^a^^,^^b^^,^^e^^,^^g^^,^^h^
Recurrence rate (4w)	3/60 (5.0%)	10/60 (16.7%)	RR 0.33 (0.11 to 1.03)	112 fewer per 1,000 (from 148 fewer to 5 more)	⨁⨁◯◯ Low^a^^,^^b^^,^^e^^,^^g^^,^^h^
Histamine	126	126	–	MD 5.76 lower (14.94 lower to 3.42 higher)	⨁◯◯◯ Very low^a^^,^^b^^,^^d^^,^^g^^,^^i^
DLQI	179	179	–	MD 3.91 lower (6.15 lower to 1.67 lower)	⨁◯◯◯ Very low^a^^,^^b^^,^^d^^,^^e^^,^^g^
IL-6	179	179	–	MD 9.34 lower (12.02 lower to 6.67 lower)	⨁◯◯◯ Very low^a^^,^^b^^,^^d^^,^^g^^,^^i^
TNF-α	119	119	–	MD 5.66 lower (8.81 lower to 2.52 lower)	⨁◯◯◯ Very low^a^^,^^b^^,^^d^^,^^g^^,^^i^
IL-4	113	113	-	MD 1.41 lower (2.02 lower to 0.79 lower)	⨁◯◯◯ Very low^a^^,^^b^^,^^d^^,^^i^
Itching score	138	138	–	MD 0.25 lower (0.43 lower to 0.07 lower)	⨁⨁◯◯ Low^a^^,^^b^^,^^e^^,^^g^^,^^i^
IgE	60	60	–	MD 1.32 SD lower (1.51 lower to 0.95 lower)	⨁◯◯◯ Very low^a^^,^^b^^,^^d^^,^^g^^,^^i^
IL-8	66	66	–	MD 6.1 lower (8.44 lower to 3.76 lower)	⨁◯◯◯ Very low^a^^,^^b^^,^^d^^,^^g^^,^^i^

a The quality of the articles is low.

b The intervention methods are different, and the dosage of the control group is not clear.

c The specific criteria for evaluating the effective rate are unknown.

d *I*^2^ > 50% may indicate substantial heterogeneity.

e Observational studies may be affected by multiple confounding factors.

f There is only one study in this group, and the number is too small.

g The index data may be biased, and the detection methods and protocols for the indicators are not specified.

h Follow-up visits for dropouts were not conducted in some articles.

i Laboratory outcome indicators are indirect.

#### 3.4.1 Fire needling monotherapy versus conventional treatment

Three studies (23–25) compared the effectiveness rate between fire needling therapy and conventional treatment at 4 weeks. No statistically significant differences were observed between groups (*p* > 0.05) across all predefined response thresholds: for effectiveness rate (>30%) [RR: 1.04 (0.90, 1.20)], effectiveness rate (>70%) [RR: 1.07 (0.84, 1.37)], or effectiveness rate (>90%) [RR: 1.30 (0.66, 2.56)].

#### 3.4.2 Fire needling therapy combined with conventional treatment versus conventional treatment alone

One study (32) compared the effectiveness rate between fire needle combination therapy and conventional treatment alone at 2-week follow-up. No statistically significant differences were found (*p* > 0.05) across all effectiveness thresholds: for effectiveness rate (>30%) [RR: 0.97 (0.86, 1.08)], effectiveness rate (>70%) [RR: 1.00 (0.74, 1.3)], or effectiveness rate (90%) [RR: 0.75 (0.43, 1.30)]. At the 4-week follow-up, no significant difference was observed between the combination therapy group and conventional treatment alone group for the >70% effectiveness rate threshold. However, for the effectiveness rate (>90%), the combination therapy group demonstrated significantly superior outcomes [RR: 2.04 (1.61, 2.59)] compared to conventional treatment alone.

For recurrence rates within 6 months, with a 2-week treatment course, one study (29) showed that the fire needling therapy combined with conventional treatment group had a lower recurrence rate than the conventional treatment alone group [RR: 0.36 (0.14, 0.93)]; with a 4-week treatment course, results from 2 studies (23, 32) showed no difference in recurrence rates between the combined treatment group and the conventional treatment alone group [RR: 0.33 (0.11, 1.03)].

For DLQI, the meta-analysis results from 3 studies (26, 29, 31) showed that the combined treatment group was significantly better than the conventional treatment alone group [MD: −3.91 (−6.15, −1.67)], and the difference was statistically significant (*p* < 0.05).

For itching score, three studies (23, 29, 30) compared the difference between fire needling therapy combined with conventional treatment and conventional treatment alone at 4 weeks. The results showed that the combined group had a lower itching score [MD: −0.25 (−0.43, −0.07)] than the conventional treatment alone group, and the difference was statistically significant (*p* < 0.05).

For inflammatory factors, the fire needle combined with conventional treatment group showed significantly lower levels of TNF-α [MD: −5.66 (−8.81, −2.52)], IL-4 [MD: −1.41 (−2.02, −0.79)], IL-6 [MD: −9.34 (−12.02, −6.77)], IL-8 [MD: −6.10 (−8.44, −3.76)], and IGE [MD: −1.23 (−1.51, −0.95)] compared to the conventional treatment alone group, with statistically significant differences (*p* < 0.05). However, no statistically significant difference was observed in histamine levels [MD: −5.76 (−14.94, 3.42)] (*p* > 0.05).

### 3.5 Safety outcomes

Adverse events were reported in three included studies (23, 27, 30), with no serious adverse events documented across all literature. Specifically, three studies involving fire needling therapy combined with topical glucocorticoids for 4 weeks reported six cases of erythema, five cases of burning sensation, two cases of hemorrhage, and two case of desquamation in the combination therapy group. Details are as follows as Table 4.

**Table 4:** Adverse events in fire needling groups.

Adverse Event	Value (*N* = 218)	Included studies
Erythema	6	(23, 27, 30)
Burning sensation	5	(23, 27)
Hemorrhage	2	(23)
Desquamation	2	(23, 30)

### 3.6 Publication bias

Due to the limited number of included studies, it was not appropriate to construct a funnel plot to assess potential publication bias.

## 4 Discussion

This meta-analysis included 10 RCTs (23–32) with a total of 868 patients with neurodermatitis, aiming to evaluate the clinical efficacy of fire needling therapy for neurodermatitis. The results suggest that fire needling therapy, especially when combined with conventional treatment, demonstrates certain advantages in treating neurodermatitis.

### 4.1 Clinical effectiveness and recurrence rate

Regarding effectiveness, fire needling monotherapy showed no statistically significant difference compared to conventional treatment. However, fire needling combined with conventional treatment demonstrated significantly better outcomes at the high efficacy threshold (>90%) after 4 weeks of treatment [RR: 2.04 (1.61, 2.59)] compared to conventional treatment alone. This suggests that fire needling therapy may enhance therapeutic effects through synergistic action with conventional treatments. Notably, after 2 weeks of treatment, the recurrence rate within 6 months was significantly lower in the fire needling plus conventional treatment group compared to the conventional treatment alone group [RR: 0.36 (0.14, 0.93)], indicating that fire needling therapy may help reduce neurodermatitis recurrence. However, with extended treatment to 4 weeks, the difference in recurrence rates between the two groups was no longer significant [RR: 0.33 (0.11, 1.03)], which may be related to limited sample size or insufficient follow-up duration.

### 4.2 Quality of life and symptom improvement

This study found that fire needling combined with conventional treatment significantly improved patients’ DLQI scores [MD: −3.91 (−6.15, −1.67)], which has important clinical implications. Neurodermatitis is primarily characterized by intense itching that severely affects patients’ sleep quality and daily activities. Our study showed that fire needling combined with conventional treatment significantly reduced pruritus scores [MD: −0.25 (−0.43, −0.07)], which may be one of the key factors in improving patients’ quality of life.

### 4.3 Changes in inflammatory cytokine levels

One important finding of this study is that fire needling combined with conventional treatment significantly reduced levels of various inflammatory cytokines, including TNF-α, IL-4, IL-6, IL-8, and IgE. These results indicate that fire needling may reduce cytokine levels, and such findings are consistent with the hypothesis that fire needling exerts therapeutic effects potentially through modulating immune function (33). Interestingly, no significant difference was observed in histamine levels between the two groups [MD: −5.76 (−14.94, 3.42)], suggesting that fire needling therapy may not primarily relieve itching symptoms by directly inhibiting histamine release but rather through other inflammatory pathways.

### 4.4 Safety assessment

Regarding adverse events, only four studies reported relevant information, mainly including local reactions such as mild erythema, burning sensation, hemorrhage, gastrointestinal reactions, and desquamation with no serious adverse events reported. These results preliminarily indicate that the safety profile of fire needling therapy appears acceptable. However, this low reporting rate of adverse events and incompleteness of safety-related information have constrained a comprehensive evaluation of the safety profile of fire needling combined with conventional treatment. Consequently, given the limited number of studies reporting adverse events, future research should pay more attention to safety assessment.

### 4.5 Limitations of the study

This meta-analysis has several important limitations. First, all included studies were conducted in China and published in Chinese, potentially introducing publication bias. Second, the overall low certainty of the evidence, as per the GRADE assessment, is an important limitation of the current evidence base; furthermore, most included studies had poor methodological quality—specifically, unclear random sequence generation methods, and the absence of allocation concealment or blinding implementation—which may affect the reliability of the results. Third, the sample sizes were relatively small, and treatment and follow-up durations were short, restrict the ability to draw firm conclusions about long-term efficacy and recurrence. Fourth, there was eterogeneity in intervention protocols and outcome measures among the included studies, which may affect the robustness of the results.

### 4.6 Clinical significance and future research directions

Despite these limitations, the findings of this study have important clinical implications. Fire needling, as a traditional Chinese medicine treatment that combines the effects of acupuncture and heat moxibustion, may exert therapeutic effects through multiple pathways, including inhibiting neurogenic inflammation, modulating immune responses, improving local microcirculation, and promoting skin barrier function restoration (34, 35). This study offers preliminary evidence-based medical support for the application of fire needling therapy in neurodermatitis treatment, particularly in aspects such as alleviating pruritus, improving quality of life, and reducing inflammatory cytokine levels—though these findings should be interpreted with consideration of their preliminary nature.

For future research, we recommend: (1) designing geographical higher-quality multicenter randomized controlled trials that strictly adher to CONSORT guidelines; (2) adopting standardized fire needling treatment protocols with clear specifications on acupoint selection, operation techniques, and treatment frequency; (3) extending follow-up periods to evaluate long-term efficacy and recurrence; (4) further exploring the mechanisms of fire needling therapy, especially its effects on skin barrier function and immune regulation.

### 4.7 Conclusion

In summary, this meta-analysis preliminarily demonstrates that fire needling therapy, especially when combined with conventional treatment, may be an effective option for treating neurodermatitis, particularly in improving pruritus symptoms, enhancing quality of life, and modulating inflammatory cytokine levels. However, given the methodological limitations of existing studies—such as unclear random sequence generation, lack of allocation concealment, regional publication bias, and small sample sizes—these factors may introduce potential biases that may skew the robustness of our conclusions. Thus, more high-quality randomized controlled trials are needed to verify these findings. We believe that pursuing multicenter trials with improved methodological rigor, longer follow-up periods, prospective registration, and mechanistic studies will be essential steps in validating and extending the preliminary findings of this analysis. Addressing these points will undoubtedly strengthen the evidence base for fire needling therapy.

## Data Availability

The original contributions presented in the study are included in the article/Supplementary material, further inquiries can be directed to the corresponding author/s.
