# Supplementary material for: Fire needling therapy for neurodermatitis: a systematic review and meta-analysis of randomized controlled trials
Source: Front Med (Lausanne). 2025 Nov 20;12:1639713. doi: 10.3389/fmed.2025.1639713 (PMC12676968; doi:10.3389/fmed.2025.1639713)
Supplement: Supplementary file 1 [file Table_1.DOCX]

**Table 1. Search strategy**

| **Database** | **No** | **Search Terms** |
| --- | --- | --- |
|  |  |  |
| **PubMed** | 1  2  3  4  5 | \| neurodermatitis[tiab] OR lichen simple chronicus[tiab] \| \| --- \| \| fire needling[tiab] OR fire needle[tiab] OR burning needle[tiab] OR hot needle [tiab] \| \| randomised controlled trial[pt] OR controlled clinical trial[pt] OR randomised[tiab] OR randomly[tiab] OR trial[tiab] OR groups[tiab] OR placebo[tiab] \| \| humans[mh] NOT animals[mh] \| \| #1 AND #2 AND #3 AND #4 \| |
| **Cochrane library** | 1  2  3  4  5  6 | (fire needle* OR fire needling OR burning needle* OR fire therapy)  (neurodermatitis OR lichen simplex chronicus)  (clinical effect* OR clinical trial* OR random* controlled study* OR placebo* OR group* allocation*)  (human* OR people OR patient*)  (animal* OR rat* OR rabbit*)  #1 AND #2 AND #3 AND #4 NOT #5 |
| **VIP** | 1  2  3  4  5  6 | \| 篇关摘：火针or烧针or火疗 \| \| --- \| \| 篇关摘：神经性皮炎 or慢性单纯性苔藓 \| \| 篇关摘：临床疗效 or 临床试验 or 随机对照研究 or 安慰 or 分组 \| \| 篇关摘：人 \| \| 篇关摘：动物 or 鼠 or 兔 \| \| #1 and #2 and #3 and #4 not #5 \| |
| **China National Knowledge Infrastructure (CNKI)** | 1 | (TI = ("火针" OR "烧针" OR "火疗") AND TI = ("神经性皮炎" OR "慢性单纯性苔藓")) OR (KY = ("火针" OR "烧针" OR "火疗") AND KY = ("神经性皮炎" OR "慢性单纯性苔藓")) OR (AB = ("火针" OR "烧针" OR "火疗") AND AB = ("神经性皮炎" OR "慢性单纯性苔藓")) AND TKA%=('临床#试验' + '临床#疗效' + '随机' + '对照' + '安慰' + '分组' + '随机对照#试验' + '随机对照#研究') AND TKA%=('人') NOT TKA%=('动物' + '鼠' + '兔') |
| **SinoMed** | 1 | (题名=(火针 OR 烧针 OR 火疗) AND 题名=(神经性皮炎 OR 慢性单纯性苔藓)) OR (关键词=(火针 OR 烧针 OR 火疗) AND 关键词=(神经性皮炎 OR 慢性单纯性苔藓)) AND NOT 题名=(动物 OR 鼠 OR 兔) AND NOT 关键词=(动物 OR 鼠 OR 兔)。 |
